# LncRNA AC098934 promotes proliferation and invasion in lung adenocarcinoma cells by combining METTL3 and m6A modifications

**DOI:** 10.7150/jca.69406

**Published:** 2022-05-16

**Authors:** Shiqing Huang, Mingyang Jin, Xiaoling Lan, Junyun Long Wu, Zhiwen Zhang, Jingjing Zhao, Yepeng Li

**Affiliations:** 1 Department of Oncology, the Affiliated Hospital of Youjiang Medical University for Nationalities, Baise, Guangxi Zhuang Autonomous Region 533000, P.R. China; 2 Department of Gynecology the Affiliated Hospital of Youjiang Medical University for Nationalities, Baise, Guangxi Zhuang Autonomous Region 533000, P.R. China; 3 Graduate School of Youjiang Medical University for Nationalities, Baise, Guangxi Zhuang Autonomous Region 533000, P.R. China

**Keywords:** lncRNA AC098934, LUAD, m6A modifications, METTL3

## Abstract

**Background:** Long non-coding RNA (lncRNA) regulates the tumorigenesis as well as the development of lung adenocarcinoma (LUAD), which is one of the high-mortality cancers. We explored the influence of lncRNA AC098934 on the malignant biological behavior of LUAD and potential underlying molecular mechanisms.

**Methods:** The expression level of AC098934 in either the LUAD or the normal tissues was identified in the TCGA database. Two AC098934 knockdown siRNAs were infected into cells of LUAD, including A549 as well as H1299 cells, using the lentivirus. Real-time Quantitative polymerase chain reaction (QPCR) helped to determine the knockdown efficiency of AC098934. CCK-8, cell cloning, wound healing combined with transwell assays tested the role of AC098934 in the cell proliferation, migration as well as the invasion. Tumor formation experiment in nude mice subcutaneously confirmed the promoting effect of AC098934 *in vivo*. In addition, combinations of METTL3 and AC098934, as well as m6A and AC098934 were identified through the RIP assay.

**Results:** Compared to the normal tissues, AC098934 was more highly expressed in LUAD tissues. After AC098934 was knocked down by siRNA, the proliferation, invasion, migration as well as tumorigenesis abilities of both A549 and H1299 cells were reduced. Mechanistically, AC098934 could bind to the m6A antibody and METTL3 protein. METTL3 overexpression promoted the m6A modification on AC098934, thereby increasing the interaction of m6A modification.

**Conclusion:** The highly expressed lncRNA AC098934 in LUAD facilitates the cell proliferation as well as invasion either *in vitro* or* in vivo*. METTL3 binds, furthermore, modulates the m6A modification of AC098934. Our research revealed a new molecular mechanism, through which AC098934 promoted the malignant behavior of LUAD tumors under the m6A modification induced by METTL3. This indicates that AC098934 is possible to be a promising biomarker as well as a therapeutic target for the patients with LUAD.

## Introduction

Cancer statistics in 2020 show that lung cancer, with high morbidity and mortality, has been the most common reason for the cancer-associated deaths worldwide, with about 135,000 persons died from it in 2020 1. As one of the two critical types in lung cancer, non-small cell lung cancer (NSCLC) is mainly constituted by lung adenocarcinoma (LUAD) 2. Although the clinical diagnosis and treatment strategies for LUAD have progressed, the prognosis of LUAD patients remains poor, especially in advanced patients due to metastasis 3, 4. Therefore, it is necessary to have further exploration of the tumorigenesis and development of LUAD, and to find new target molecules and treatment methods.

Long non-coding RNAs (lncRNAs) are constituted by more than 200 bases, with a cap structure at the 5'end as well as a poly (A) tail at the 3'end, but they do not encode proteins. With the continuous deepening of research, the role of lncRNA in physiology and disease has received more and more attention 5, 6. Abnormal expression of lncRNA has been found in tumors, cardiovascular and neurodegenerative diseases 7-9. LncRNAs can be used as precursors of microRNA, or sponge molecules, to adsorb microRNA and hence participate in the regulation of target genes 10. LncRNAs also affect the stability of the target protein by binding to it, thereby regulating related signaling pathways 11. Previous studies have confirmed that lncRNA MALAT1, HOXA11, SNHG7, DLG2 and UPLA1 regulated the occurrence as well as the progression of LUAD. Besides, they possibly acted as the diagnosing biomarkers as well as treatment targets 12-16. However, the function of other lncRNAs in LUAD is not clear.

N6-methyladenosine (m6A) is the most abundant epi-transcriptomic modification in eukaryotic mRNA. Studies have shown that abnormal m6A RNA methylation occurs in the formation and development of a variety of tumors, including LUAD, and affects tumor proliferation, apoptosis, metastasis, drug resistance and immunosuppressive processes 17, 18. M6A RNA methylation depends on the METTL3/METTLI4/WTAP complex, which performs methylase activity and function 19. Studies have shown that some non-coding RNAs, including lncRNA, such as MALAT1 and LCAT3, regulated the m6A RNA methylase complex and participated in the progression of lung cancer 18, 20. Therefore, m6A-modified regulatory lncRNA has a potential as the molecular target for tumor diagnosis as well as treatment.

Recently, a new type of lncRNA, lncRNA AC098934, has been discovered. Based on the results of studies, lncRNA AC098934.2-201 isoform, also known as AGPG, was involved in the process of tumor glycolysis and was a predictor for the malignant transformation of esophageal cancer 21, 22. The present study suggests that AC098934 exerts an important influence on LUAD, and confirms that AC098934 participates in the development of LUAD based on the m6A methylation pathway.

## Materials and Methods

### Cell lines and cell culture

LUAD cell lines, including A549, H1299, HCC827, H226, H23 cells, and normal bronchial epithelial cells (BEAS-2B) were purchased from American Type Culture Collection (ATCC, Manassas, VA, USA), followed by the cell culture in a 5% CO_2_ incubator (37°C) in 1640 medium (Gibco, NY, USA) having 10% fetal bovine serum (FBS) (Gibco, NY, USA).

### Bioinformatics Analysis

The gene expression profiles of 483 LUAD patients and 347 normal samples were downloaded from the TCGA database (https://cancergenome.nih.gov/). The inclusion criteria of patients enrolled was pathological diagnosed as lung adenocarcinoma and without any treatment as surgery, radiation therapy, chemotherapy or target therapy. All major histologic types of lung adenocarcinoma were represented including lepidic, acinar, papillary, micropapillary, solid, invasive mucinous, colloid and unclassifiable adenocarcinoma 23, 24. The analysis of AC098934's RNA sequencing data between LUAD and normal tissues was performed by R language (version 4.0) and the edgeR package was used for the analysis of gene differential expression and survival proportion. The value of P <0.05 was considered to be statistically significant.

### RNA interference and lentiviral vectors

The sequence of two AC098934 siRNAs was inserted into the lentiviral vector hU6-MCS-CBh-gcGFP-IRES-puromycin (LVKL20263, Genechem, Shanghai, China, https://www.genechem.com.cn) to construct a stable AC098934 knockdown vector. The siRNA sequence of AC098934 is shown in Table 1. Two vectors containing AC098934 siRNA were packaged into lentivirus and then infected into A549 and H1299 cells according to the lentivirus cell infection manual from Genechem. 2 μg over-express plasmid targeting METTL3 or AC098934 in 100 μl RMPI 1640 was mixed with 2 μL lipofectamine 3000 (Invitrogen, Calsbad, CA, USA) diluted in 100 μL RMPI 1640 for 20 mins at room temperature according to the manufacturer's instruction. The cells were collected 48 h after transfection.

### Real-time Quantitative polymerase chain reaction (QPCR)

Normal and AC098934-knockdown (KD) A549/H1299 cells were lysed with 500 μL Trizol reagent, and RNA precipitated with 75℅ ethanol. Reverse transcription from RNA to cDNA was carried out by RevertAid Reverse Transcriptase (EP0441, Thermo, USA) according to manufacturer's instruction. Gene expression in each cDNA sample was performed complying with the instructions of the manufacturer for PerfectStart Green qPCR SuperMix (AQ601-04, TransGen Biotech, China). The RT-PCR primers used to identify AC098934 in the cell nuclei and AC098934-KD efficiency are listed in Table 2. For either A549 or H1299 cells, the cytoplasmic and nuclear components were separated complying with the instructions by the manufacturer (P0027, Beyotime Biotech, China). Reaction conditions for PCR were: 94˚C for 10 min (Denaturation of starting template), 40 cycles: 95˚C for 5 s, 60˚C for 15 s and 72˚C for 10 s. The relative expression levels of AC098934 were calculated by 2^-ΔΔCt^ with GAPDH as internal reference.

### Cell Counting Kit-8 (CCK-8)

For A549, H1299 and H226 cells, CCK-8 (Beyotime, China) was adopted to assess their proliferation. Firstly, the AC098934-KD cells and normal LUAD cells were trypsinized, followed by the seeding with 8,000 cells per well in the 96-well plate. Then CCK8 detection was performed after 1-5 days. For detection, CCK-8 solution (10 μL) was placed into each well. After 1 h incubated (37℃, 5% CO2), the measurement of the absorbance value was performed at 450 nm.

### Apoptosis measurement based on flow cytometry

Treated A549, H1299 and H226 cells were firstly washed with pre-cooled saline, followed by the resuspension in binding buffer (500 μL) to adjust the concentration to 1×10^7^/mL. According to the manufacturer's kit (640914, Biolegend, USA) instructions, cell suspension (100 μL) was firstly mixed with Annexin V-FITC (5 μL) plus Propidiμm Iodide (5 μL), followed by the incubation in the dark at room temperature for 15 min. Subsequently, CytoFLEX flow cytometer (BECKMAN, USA) was adopted to detect the cell apoptosis. And the data was analyzed by Cytexpert 2.0 ((Beckman Coulter).

### Colony formation assay

Colony formation experiment was designed to assess the ability of cell proliferation among single cells after A549 and H1299 were transfected with different siRNAs. After seeded on a 6-well cell culture plate (400 cells per well), cells infected with lentivirus were cultured in a cell culture incubator for 10-14 days. After fixed in 4% paraformaldehyde (PFA) for 20 min, cell clones received crystal violet staining for 10 min. Subsequently, those on the dried cell plate were photographed, and the number was counted manually.

### Wound healing assay

Treated A549 and H1299 cells were seeded and reproduced in a 6-well culture plate until the density reached 90%. After 24 h, a wound was scratched with the tip of a micropipette. Photographs of the cells were taken at the very beginning as well as 24 h of incubation.

### Transwell invasion assay

After diluted in serum-free medium, Matrigel (100 μL) was put into the transwell chamber. The cells of each group were resuspended in serum-free medium, followed by the addition of 300 μL of cell suspension (1×10^5^) to the chamber. The medium (500 μL) having 10% FBS was laid outside of the transwell chamber, afterwards, incubated for 24 h in a cell incubator. Subsequently, the fixation of cells with 4% PFA was carried out for as long as 20 min, among which those not invading the basal membrane inside the invasion chamber were discarded. Subsequently, the outside of the transwell chamber was dyed for 10 min with crystal violet. Finally, the photograph of the invaded cells were took by an optical microscope (Ts2-FL, Nikon, Japan), with the number of invaded cells counted for each group. The invaded cells were manually counted from 10 random fields at x100 magnification.

### Nude mice xenograft tumor experiment

Eighteen nude mice in all, male and aged 4-5 weeks, were obtained and placed in Hunan Slack Jingda Experimental Animal Co., Ltd., afterwards, divided into three groups in random. A549 cells expressing negative control siRNA (NC-siRNA) and AC098934-siRNA were injected subcutaneously into nude mice at 1×10^7^ in a 100 μL volume. Nude mice were sacrificed 21 days later, with the size as well as the weight of tumors in each group recorded.

### RNA immunoprecipitation

A549 together with H1299 cells (1×10^7^) were resuspended in the buffer of RNA immunoprecipitation (RIP). Then 10 µg of m6A antibody (ab208577, Abcam) or MELLT3 antibody (ab195352, Abcam) were added to the cell lysis supernatant and incubated overnight at 4°C, afterwards, the addition of 40 µL protein A/G magnetic beads (Bimake, China) and 1h of incubation was carried out. The magnetic beads were firstly washed and then resuspended with RIP buffer (500 µL). Finally, the level of AC098934 RNA bound to the magnetic beads was detected by RT-PCR.

### Statistical Analysis

All the statistical analysis in this study was carried out with Graphpad Prism 8 (GraphPad Software, Inc.). Comparisons between two groups was conducted using two tailed unpaired Student's t-test or one-way ANOVA with Tukey's as post hoc test for multiple group comparisons. The value of P< 0.05 was regarded to be statistically significant difference.

## Results

### The clinical analysis of AC098934 and METTL3

**Figure 1:**
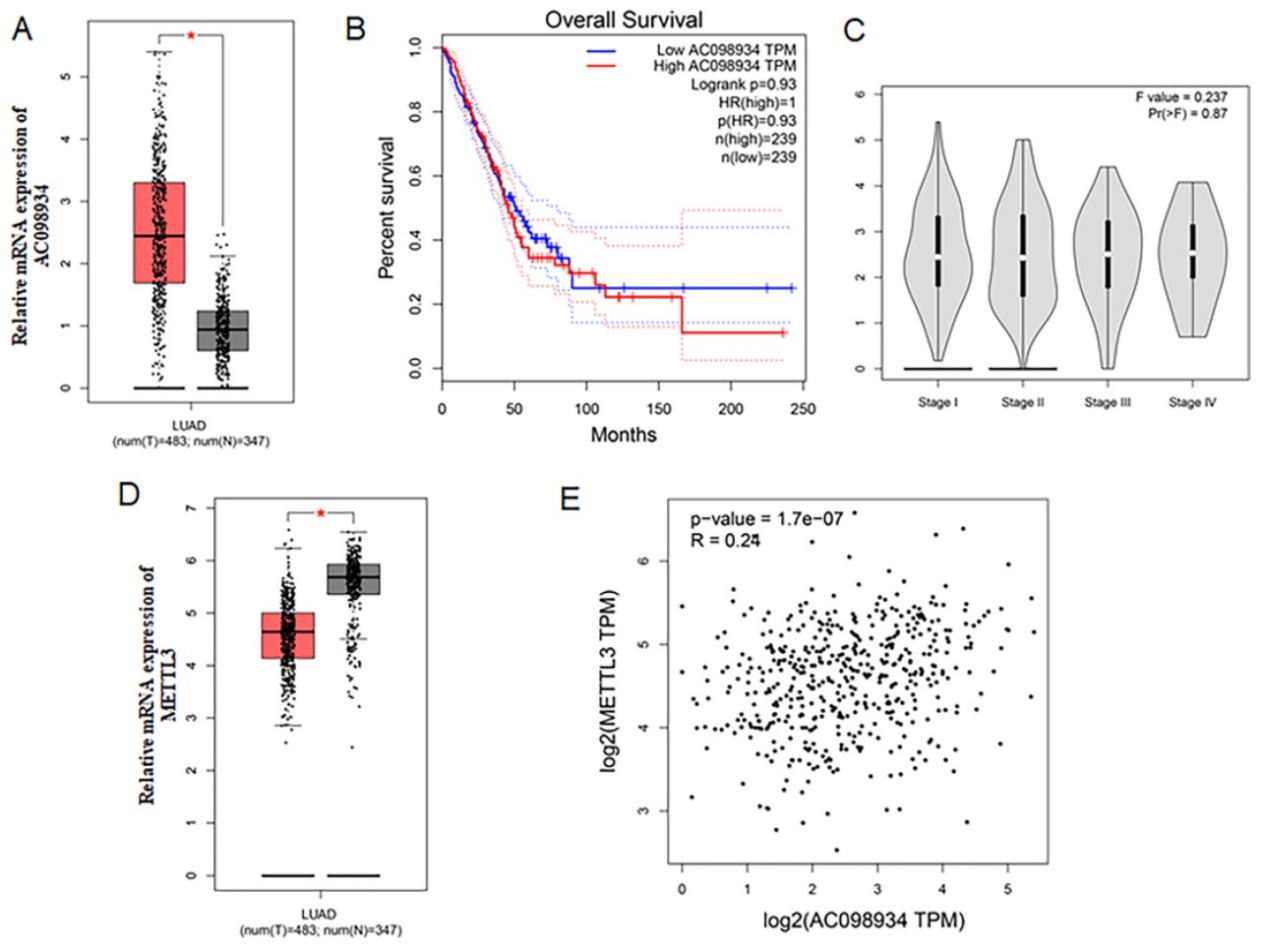
The clinical analysis of AC098934 and METTL3 in the TCGA dataset. A. The expression of AC098934 in cancer and normal tissues. ^*^P<0.05. B. The overall survival of LUAD patients with different expression level of AC098934. C. The expression of AC098934 in LUAD patients in different clinical stages. D. The expression of METTL3 in cancer and normal tissues. ^*^P<0.05.E. The correlation of AC098934 and METTL3 in LUAD patients. R=0.24, P<0.0001.

Firstly, The LUAD data set from TCGA was analyzed. Compared to the normal tissues, AC098934 was more highly expressed among LUAD tissues (n = 483) (Figure 1A). The overall percent survival of LUAD patients with lower AC098934 expression had no difference with those with higher AC098934 expression level (Figure 1B), which indicates that AC098934 may be involved in the process of tumor initiation and tumor maintenance. And AC098934 level didn't change with the clinical stage of LUAD patients (Figure 1C). METTL3 expression level decreased in LUAD tissues compared with the normal tissues (Figure 1D). The correlation analysis demonstrated that AC098934 expression had a low correlation 25 with METTL3 expression (R^2^=0.0576) (Figure 1E). The analysis for the LUAD data set from TCGA showed AC098934 may be involved in the tumorigenesis and tumor maintenance process.

### The decreased expression of AC098934 inhibits the cell proliferation in LUAD cells (A549 and H1299)

**Figure 2:**
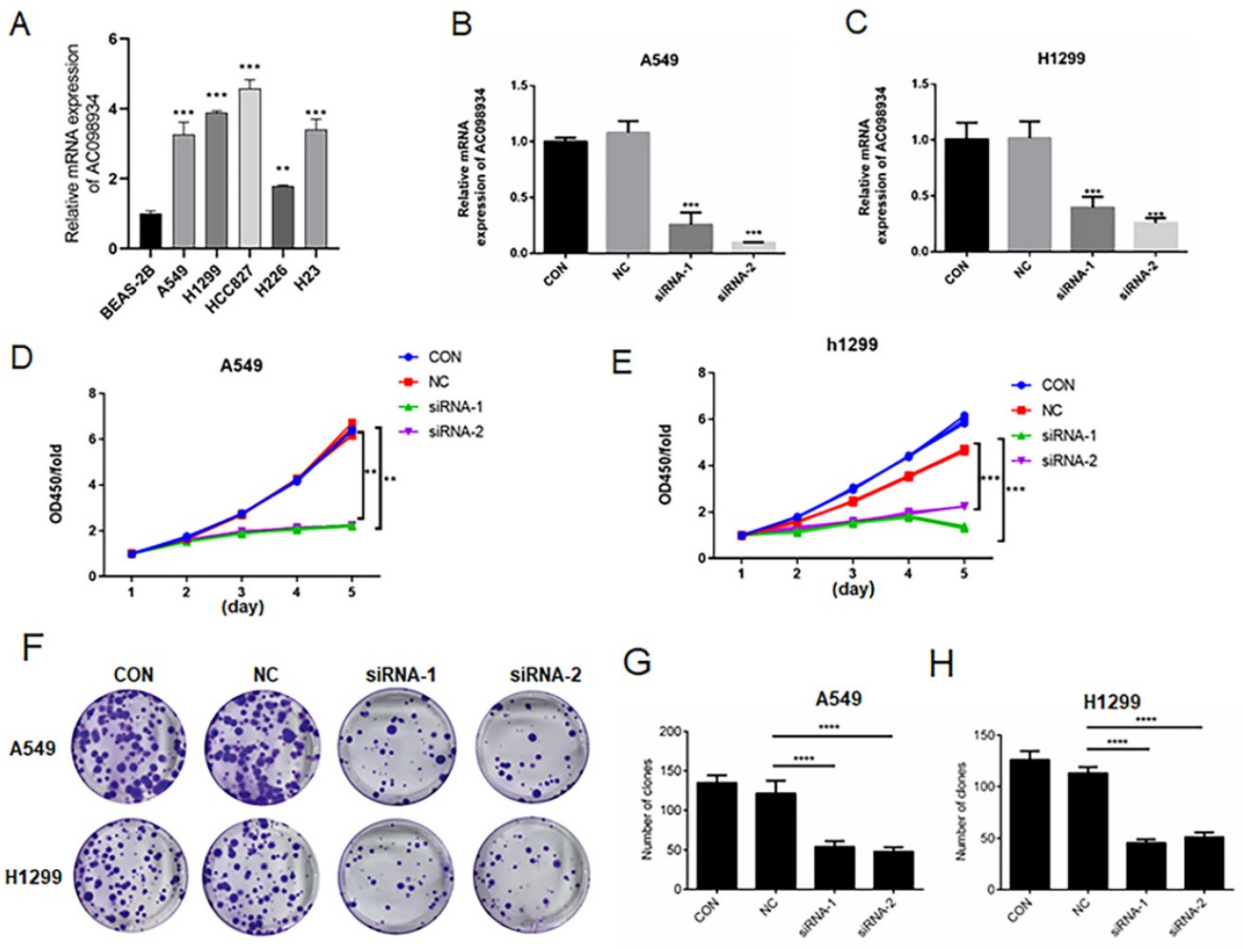
The decreased expression of AC098934 inhibits the cell proliferation in LUAD. A. The relative mRNA expression of AC098934 in a normal bronchial epithelial cell and five LUAD cell lines. B.C. RNA from normal control A549 or H1299 cells (CON), cells expressing negative control siRNA (NC) and AC098934-targeting siRNA-1, siRNA-2 was extracted. The interference effect on AC098934 was identified by RT-PCR. ^***^P<0.001. D.E. CCK-8 assay was used to detect the cell proliferation of A549 or H1299 cells in each group within 5 days. ^**^P<0.01, ^***^P<0.001. F. Normal control (CON), negative control siRNA (NC) and AC098934 siRNA (siRNA-1 and siRNA-2) were transferred into A549 or H1299 cells. The typical colony dot pattern of each group of cells is displayed. H.I. For either A549 or H1299 cells, the number of clones under each treatment was counted. ^****^ P<0.0001.

In five LUAD cell lines, we selected A549 and H1299 cells as highly expressed research models (Figure 2A). We introduced siRNA targeting AC098934 into the LUAD cell lines in human, including A549 cells together with H1299 cells. Through RT-PCR, we tested the knockdown efficiency of two siRNAs targeting AC098934 (Figure 2B and C). This confirmed that two specific siRNAs helped to reduce the expression level of AC098934 in the LUAD cell lines. We next examined the role of AC098934 in the proliferation of LUAD cells. We performed AC098934-siRNA transfer on cultured A549 and H1299 cells, and detected the proliferation of the cells by means of the CCK-8 assay. According to the results, cell proliferation in A549 (Figure 2D) as well as H1299 (Figure 2E) were significantly more inhibited by the two AC098934-siRNAs when compared to normal control cells (CON) and negative control siRNA (NC).

In addition, knocking down AC098934 (Figure 2F) inhibited the colony formation of A549 cells (Figure 2G) as well as H1299 cells (Figure 2H). Our data confirms that lncRNA AC098934, with highly expression in LUAD, promotes the cell proliferation.

### The decreased expression of AC098934 reduces invasion, metastasis and apoptosis in LUAD cells

**Figure 3:**
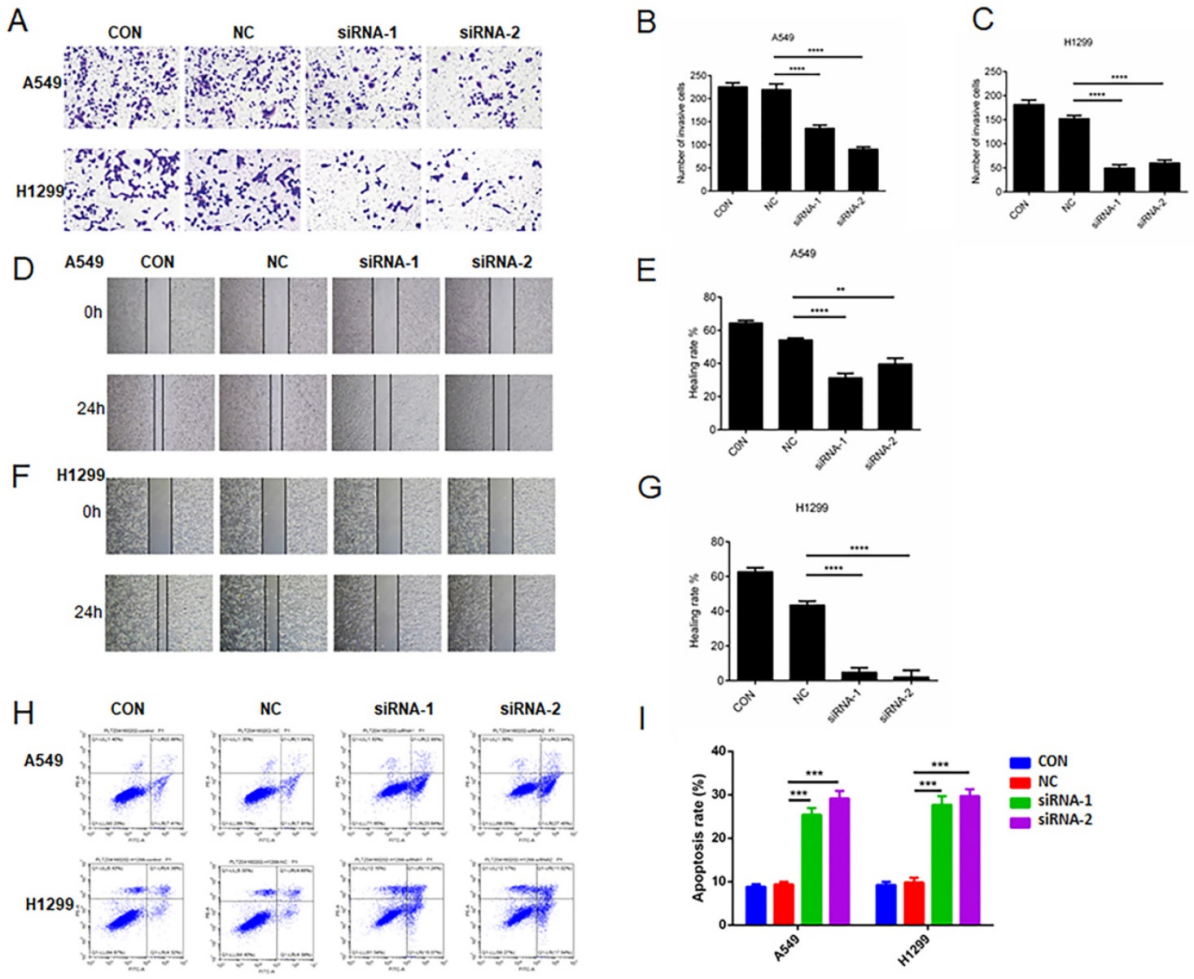
The decreased expression of AC098934 reduces invasion/metastasis and apoptosis in LUAD cells. A. The representative image of transwell invasion experiment showed the morphology and number of invaded A549 or H1299 cells under each treatment. B.C. For either A549 or H1299 cells, the number of invaded under each treatment was counted. ^****^ P<0.0001. D.F. In the wound healing experiment, the cell migration images of A549 or H1299 cells under each treatment at 0 and 24 hours were shown. E.G. The percentage of healing or migration of A549 or H1299 cells under each treatment was counted. ^**^ P<0.01, ^****^ P<0.0001. H. Flow cytometry was used to detect the proportion of apoptotic cells in A549 or H1299 cells expressing negative control siRNA (NC) and AC098934 siRNA (siRNA-1 and siRNA-2). I. The percentage of apoptotic cell population of A549 or H1299 cells under each treatment was counted. ^***^ P<0.001.

We seeded A549 or H1299 cells expressing NC-siRNA and two AC098934-siRNAs in the transwell plate (Figure 3A). The number of invading and migrating A549 or H1299 cells expressing two AC098934-siRNAs was significantly reduced compared with NC-siRNA (Figure 3B and C). Then, we used the wound healing assay to check whether the migration ability of LUAD cells changed 24 h after AC098934 was knocked down. Compared with NC-siRNA, the healing rate of A549 (Figure 3D and E) or H1299 cells (Figure 3F and G) expressing AC098934-siRNA was significantly reduced. Therefore, the expression of lncRNA AC098934 enhances LUAD cell metastatic potential. Flow cytometry data showed the proportion of apoptotic cell populations in A549 as well as H1299 cells (Figure 3H). Compared to the NC-siRNA, the proportion of apoptotic cells among the LUAD cells knocked down by AC098934-siRNA increased and the proportion of surviving cells decreased (Figure 3I). In LUAD cells with highly expressed AC098934, lncRNA AC098934 tends to promoting cell invasion and metastasis, inhibit cell apoptosis, thereby promoting cell invasion and growth.

### The increased expression of AC098934 promotes the cell proliferation, invasion, and apoptosis in LUAD cells (H226)

**Figure 4:**
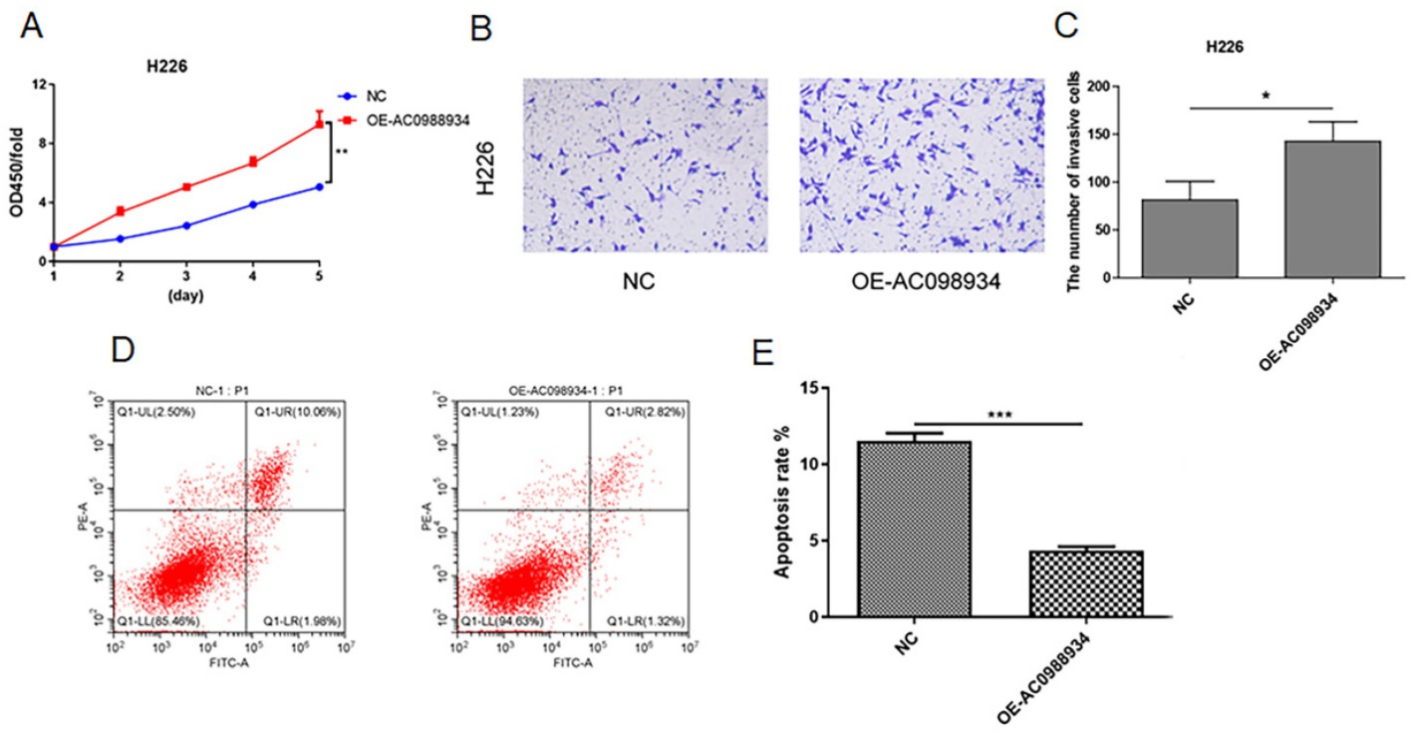
The increased expression of AC098934 promotes the cell proliferation, invasion and apoptosis in LUAD cells (H226). A. CCK-8 assay was used to detect the cell proliferation of H226 cells in each group within 5 days. ^**^P<0.01. B.C. The representative image of transwell invasion experiment showed the morphology and number of invaded H226 cells under each treatment. ^*^P<0.05. H. Flow cytometry was used to detect the proportion of apoptotic cells in H226 cells expressing negative control siRNA (NC) and OE-AC098934. I. The percentage of apoptotic cell population of H226 cells under each treatment was counted. ^***^ P<0.001.

In five LUAD cell lines, H226 cells was more lowly expressed research models (Figure 2A). We introduced over-expression plasmid targeting AC098934 (OE-AC098934) into the H226 cells in human. Then we detected the proliferation of the cells using the CCK-8 assay. According to the results, cell proliferation in H226 (Figure 4A) was significantly more promoted by the OE-AC098934 when compared to negative control (NC). Then we performed the transwell test (Figure 4B). The number of invading H226 cells in AC098934 over expressed group was significantly increased compared with NC group (Figure 4C). Flow cytometry assay showed the rate of apoptotic cell populations in H226 cells (Figure 4D). Compared to the NC, the rate of apoptotic cells among the H226 cells with over expressed AC098934 reduced and the rate of surviving cells increased (Figure 4E). In LUAD cells with lowly expressed AC098934, lncRNA AC098934 tends to promote proliferation and invasion, inhibit cell apoptosis, thereby promoting cell growth and invasion.

### AC098934 enhances tumorigenesis in a LUAD mouse model

**Figure 5:**
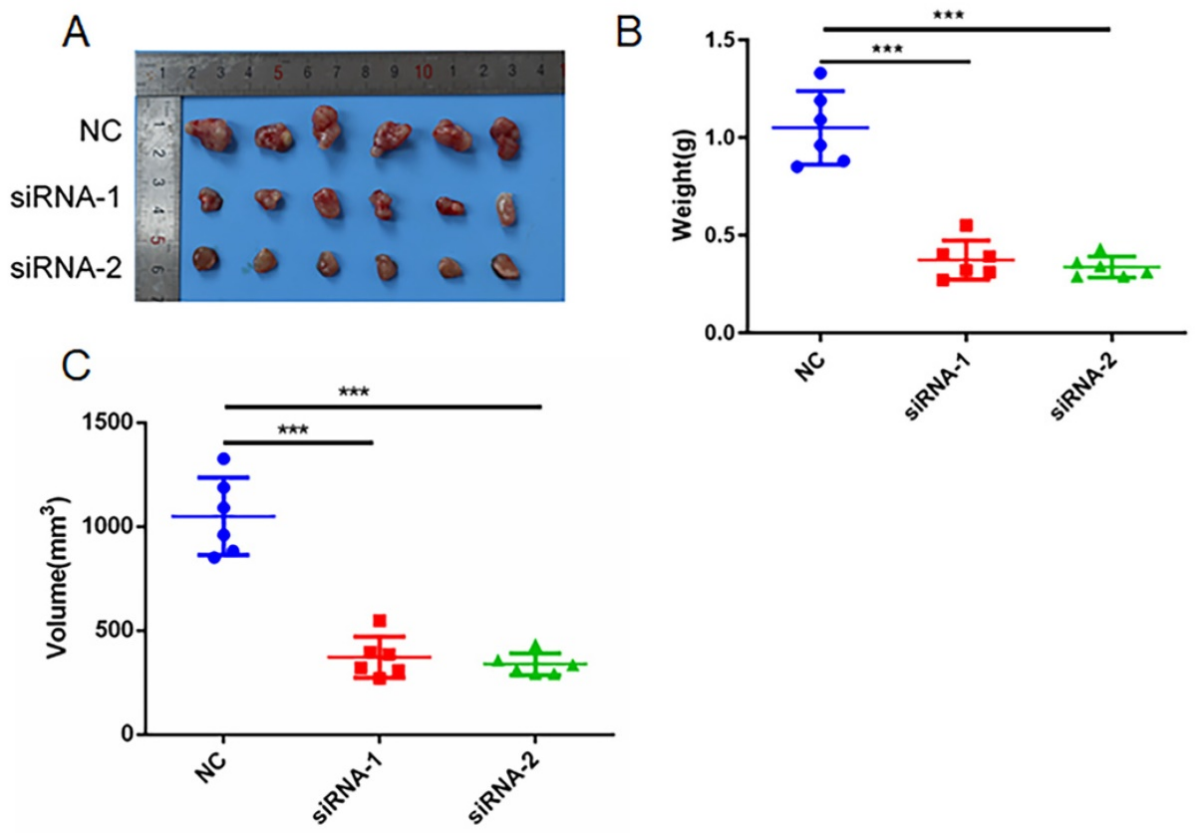
AC098934 enhances LUAD cell tumor growth *in vivo*. A. A549 cells expressing negative control siRNA (NC) and AC098934 siRNA (siRNA-1 and siRNA-2) were subcutaneously grown in nude mice. B. The weight of tumors expressing NC, siRNA-1 and siRNA-2 was counted. C. The volume of tumors expressing NC, siRNA-1 and siRNA-2 was recorded. ^***^ P<0.001.

Next, we studied the function of AC098934 in LUAD tumor formation *in vivo*. We conducted subcutaneous tumorigenic experiments on A549 cells transfected with NC-siRNA or AC098934-siRNA in nude mice. We found that A549 cells expressing AC098934-siRNA had significantly reduced tumorigenesis ability in nude mice (Figure 5A). The tumor mass (Figure 5B) and size (Figure 5C) of the subcutaneous tumors of nude mice expressing AC098934-siRNA were significantly smaller than the NC-siRNA group. These results indicate that AC098934 promotes LUAD tumorigenesis *in vivo*.

### METTL3 regulates the m6A modification of AC098934 in LUAD cells

**Figure 6:**
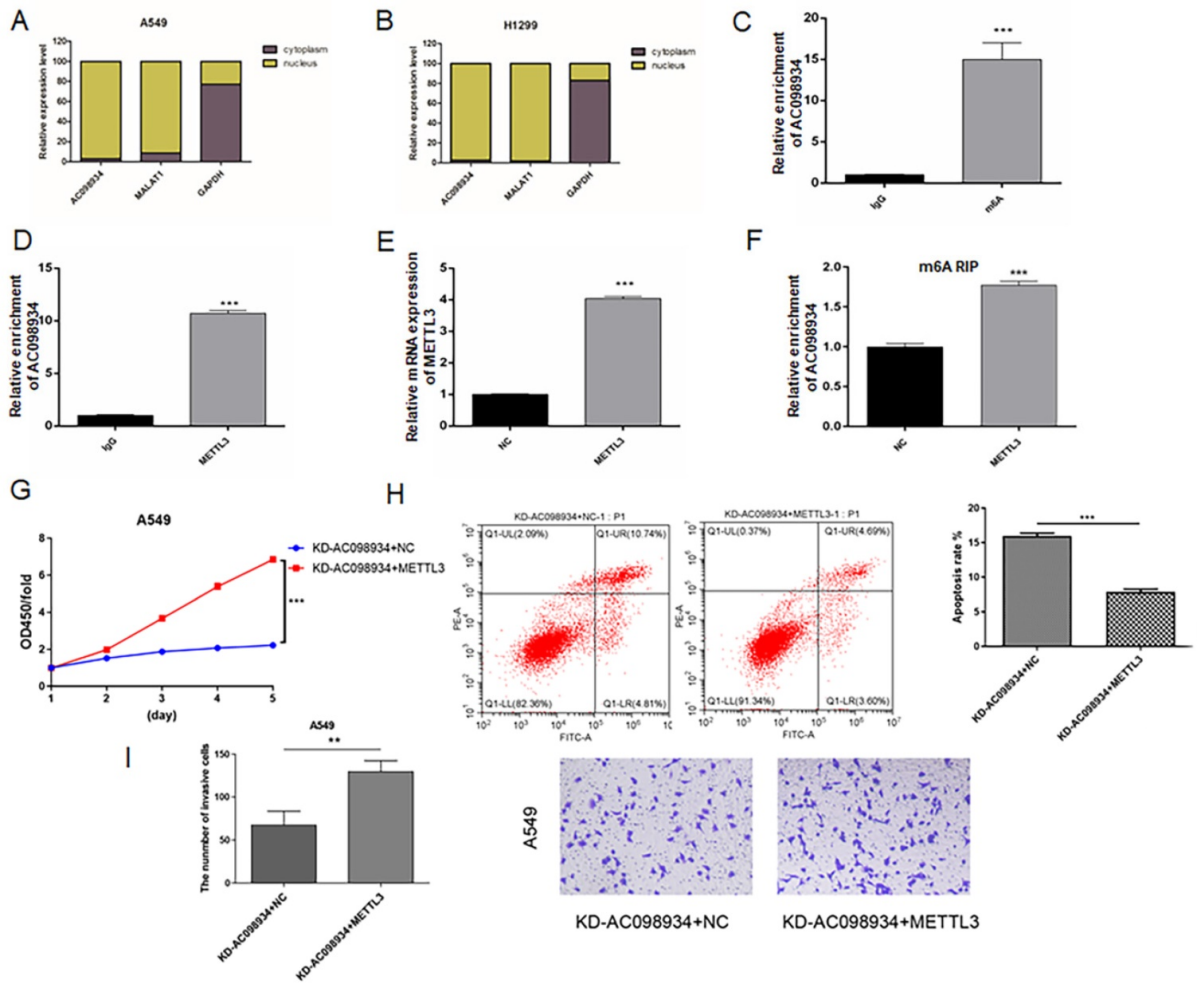
AC098934 is modified by m6A and interacted with METTL3. A.B. The expression level of AC098934 in the cytoplasm as well as nucleus of A549 or H1299 cells was measured by RT-PCR. C. The RNA level of AC098934 bound to m6A was detected by RIP enrichment assay. ^***^ P<0.001. D. The RNA level of AC098934 bound to METTL3 was detected by RIP enrichment assay. ^***^ P<0.001. E. METTL3 overexpression plasmid together with NC vector was transfected into A549 cells. Besides, mRNA level of METTL3 between the two groups of cells was determined using RT-PCR. ^***^ P<0.001. F. RIP enrichment assay was performed to detect the RNA level of AC098934 bound by m6A in METTL3 overexpression cells and control A549 cells. ^***^ P<0.001. G.H.I. Rescue assay to validate the functions of AC098934 in promoting proliferation and invasion via METTL3. ^**^ P<0.01, ^***^ P<0.001.

To further study the mechanism of AC098934 in LUAD cells, we firstly isolated the cytoplasmic as well as nuclear components of either A549 or H1299 cells, afterwards, identified the RNA location of AC098934 by RT-PCR. It was found that the location of AC098934 was mostly inside the nucleus of A549 (Figure 6A) or H1299 cells (Figure 6B). As a positive control, the localization of lncRNA MALAT1 (which has been proven to promote the LUAD process in the nucleus 26) in the nucleus, and the localization of GAPDH mRNA in the cytoplasm were confirmed. Furthermore, we found that AC098934 was modified by m6A in the nucleus of LUAD cells.

We performed RNA immunoprecipitation (RIP) experiments using an m6A antibody. It was found that, compared to the IgG antibody group, the relative enrichment expression of AC098934 within the m6A antibody group was significantly more increased (p<0.001, Figure 6C). The result suggested that there was an m6A site on AC098934, which could bind to m6A antibody. Besides, the results of RIP enrichment of METTL3 antibody indicated that compared to the IgG antibody group, the relative expression of AC098934 enriched by METTL3 antibody was significantly higher as well (p<0.001, Figure 6D), which demonstrated AC098934 RNA could bind to METTL3 protein. We transfected the METTL3 overexpression vector and the NC vector into A549 cells (Figure 6E), and performed a RIP assay with m6A antibody, discovering that compared to the NC cells, the relative expression of AC098934 enriched with m6A antibody in METTL3 overexpressing cells was significantly more increased (p<0.001, Figure 6F). Based on our data, it was suggested that AC098934 located in the nucleus has m6A modification sites, which bind to m6A and METTL3. METTL3 overexpression promoted the m6A modification of AC098934, thereby enhancing its interaction with the m6A antibody.

To validate the AC098934's biological function in LUAD cells via METTL3, a rescue assay was performed. AC098934 siRNA and or METTL3 over expression plasmid were infected with A549 cells. Cells with AC098934 siRNA and negative control of METTL3 (KD-AC098934+NC) showed lower proliferation (Figure 6G), higher apoptosis proportion (Figure 6H) and less invasive cells (Figure 6I) compared to the cells transfected with both AC098934 siRNA and over expressed METTL3 vector (KD-AC098934+METTL3). The data suggested that knocking down of AC098934 inhibited the cell growth and invasion in LUAD cells, which could be rescued by over expression of METTL3, which increase the m6A modification of AC098934.

## Discussion

LUAD is the main histological subtype in NSCLC (approximately 40-50%). Currently, most of the LUAD patients have reached the advanced stage with tumor invasion and metastasis when diagnosed 27, 28. This is the main factor resulting in the poor prognosis. Therefore, in-depth exploration into the molecular mechanism underlying the pathogenesis of LUAD has important clinical significance for finding potential therapeutic targets.

The malignant growth and metastasis of LUAD cells are controlled by specific gene expression profiles and biomarkers. These genes or proteins play a crucial regulatory role in the pathology of LUAD 29. Nowadays, lncRNA has been found to induce tumors through a variety of mechanisms, and affects tumor growth and invasion 30. LncRNA also exerts an important regulatory effect on the occurrence as well as the development of lung cancer and even LUAD. Through sequencing or online databases such as TCGA, researchers have screened a series of lncRNA that regulate the biological behavior of LUAD and revealed their role in miRNA-mRNA regulation 31. Transcriptomics were also used to screen new lncRNA and their abnormal expression in the pathology and treatment of LUAD 32. Specifically, some unique effects of lncRNA on LUAD have been revealed. For example, lncRNA DGCR5 reduced the expression of hsa-mir-22-3p and promoted LUAD tumor growth 33. The upregulation of LncRNA MACC1-AS1 in LUAD cells and tissues was negatively associated with the expression of PTEN 34. For cisplatin-resistant LUAD cell lines, LncRNA activated by transforming growth factor-beta (ATB) was obviously up-regulated via the miR-200a or beta-catenin pathway 35. The above studies have confirmed that lncRNA exerts an influence to the proliferation, invasion, metastasis and apoptosis of the cells in lung cancer. Our research reveals a new type of lncRNA called AC098934. In LUAD, AC098934 facilitated the cell proliferation, migration as well as invasion in tumors. Our data also reveals that AC098934 knockdown can inhibit the growth of LUAD *in vivo*, thereby providing a new strategy for the treatment of LUAD.

The mechanism of AC098934's malignant behavior on LUAD may depend on the m6A modification of AC098934, which is confirmed by our data. The m6A methylation modification of RNA (including mRNA, lncRNA and other non-coding RNA) extensively regulates physiological activities of tumor cells 36, 37. The abnormal expression of m6A-related proteins leads to the malignant proliferation, metastasis and drug resistance in lung cancer cells or LUAD. The m6A reader YTHDF2 bound to the untranslated region of 6-phosphogluconate dehydrogenase mRNA, and promoted the growth of tumor cells as well as upregulated the pentose phosphate pathway (PPP) 38. The m6A demethylase FTO increased the stability of the ubiquitin-specific protease 7 mRNA and reduced the m6A level, which induced the growth of NSCLC cells 17. Among these m6A modified proteins, the m6A methyltransferase METTL3 is at the core of the methyltransferase complex. The risk prediction model established based on the online database showed that the m6A methylation regulation mediated by METTL3 was related to the overall survival of LUAD cells 39. METTL3 expression was elevated in LUAD tissues, and was associated with ribosomes to promote the translation of various mRNAs in the cytoplasm 40. Knockdown of METTL3 inhibited the TGF-β-induced epithelial-mesenchymal transition (EMT) of lung cancer cells 41. Our results confirmed that METTL3 binds AC098934 and promotes its m6A modification, which may be responsible for LUAD cell malignant proliferation.

In fact, lncRNA is bound up with the m6A methylation pathway, assisting lung cancer progress. For example, lncRNA LCAT3 was modified by METTL3-mediated m6A modification, which activated MYC transcription to facilitate LUAD cell's proliferation 20. ALKBH5 demethylated the m6A of lncRNA RMRP and promoted tumorigenesis of LUAD 42. METTL3 installed and enhanced the m6A modification of lncRNA ABHD11-AS1, which promoted the proliferation of NSCLC and the Warburg effect 43. Previous study also proved that AC098934 mediated tumor abnormal glycolysis and the regulation of the Warburg effect 21. Furthermore, our data adds to the mechanism by which AC098934 affects tumor development, by highlighting the role of the m6A modification mediated by METTL3. We suggest that overexpression of METTL3 increases AC098934's m6A modification and binding to m6A antibody. Therefore, AC098934 as a new target molecule is expected to become a biomarker of LUAD.

## Figures and Tables

**Table 1:** The siRNA-1 and siRNA-2 sequence of AC098934

NO.	5'	STEM	Loop	STEM	3'
AC098934-RNAi(siRNA-1)-a	Ccgg	CAGATGGGCAATGCCTGCTGG	CTCGAG	TAGCAACAAATGTTTCAGCTG	TTTTTg
AC098934-RNAi(siRNA-1)-b	aattcaaaaa	CAGATGGGCAATGCCTGCTGG	CTCGAG	TAGCAACAAATGTTTCAGCTG	
AC098934-RNAi(siRNA-2)-a	Ccgg	GGGAGACCCAGCCTGGCAGGC	CTCGAG	TAAATTGCTTCCTCTTTGCCC	TTTTTg
AC098934-RNAi(siRNA-2)-b	aattcaaaaa	GGGAGACCCAGCCTGGCAGGC	CTCGAG	TAAATTGCTTCCTCTTTGCCC	

**Table 2:** Primers for q-PCR of AC098934

Primers	Sequencing (5'→3')
has- AC098934-F	GTTGGGATTCGTGGTGTTGC
has- AC098934-123-R	TGCCAACCTATGACAGCAGG
GAPDH-127F (H)	CCAGGTGGTCTCCTCTGA
GAPDH-127R (H)	GCTGTAGCCAAATCGTTGT
**Primers for identification in cell nuclei**	
AC098934-169-F	AGAGGGCAGAAGGGAAGTTC
AC098934-169-R	TGATGAAGGGCCACAGGAAT
hMALAT1-F	AAAGCAAGGTCTCCCCACAAG
hMALAT1-R	GGTCTGTGCTAGATCAAAAGGCA
h-GAPDH-168-F	AGGTCGGAGTCAACGGATTT
h-GAPDH-168-R	TGACGGTGCCATGGAATTTG

## References

[1] Siegel RL, Miller KD, Jemal A (2020). Cancer statistics, 2020. CA Cancer J Clin.

[2] Sanchez-Cespedes M, Parrella P, Esteller M (2002). Inactivation of LKB1/STK11 Is a Common Event in Adenocarcinomas of the Lung. Cancer Research.

[3] Popper HH (2016). Progression and metastasis of lung cancer. Cancer Metastasis Rev.

[4] Altorki NK, Markowitz GJ, Gao D (2019). The lung microenvironment: an important regulator of tumour growth and metastasis. Nat Rev Cancer.

[5] Peng WX, Koirala P, Mo YY (2017). LncRNA-mediated regulation of cell signaling in cancer. Oncogene.

[6] Jathar S, Kumar V, Srivastava J (2017). Technological Developments in lncRNA Biology. Adv Exp Med Biol.

[7] Bhan A, Soleimani M, Mandal SS (2017). Long Noncoding RNA and Cancer: A New Paradigm. Cancer Res.

[8] Huang Y (2018). The novel regulatory role of lncRNA-miRNA-mRNA axis in cardiovascular diseases. J Cell Mol Med.

[9] Riva P, Ratti A, Venturin M (2016). The Long Non-Coding RNAs in Neurodegenerative Diseases: Novel Mechanisms of Pathogenesis. Curr Alzheimer Res.

[10] Han TS, Hur K Epigenetic Associations between lncRNA/circRNA and miRNA in Hepatocellular Carcinoma. 2020; 12(9): 2622.

[11] Ferrè F, Colantoni A, Helmer-Citterich M (2016). Revealing protein-lncRNA interaction. Brief Bioinform.

[12] Chen J, Liu X, Xu Y (2019). TFAP2C-Activated MALAT1 Modulates the Chemoresistance of Docetaxel-Resistant Lung Adenocarcinoma Cells. Mol Ther Nucleic Acids.

[13] Zhao X, Li X, Zhou L (2018). LncRNA HOXA11-AS drives cisplatin resistance of human LUAD cells via modulating miR-454-3p/Stat3. Cancer Sci.

[14] Pei YF, He Y, Hu LZ (2020). The Crosstalk between lncRNA-SNHG7/miRNA-181/cbx7 Modulates Malignant Character in Lung Adenocarcinoma. Am J Pathol.

[15] Arenas AM, Caudros M Andrades A (2020). LncRNA DLG2-AS1 as a Novel Biomarker in Lung Adenocarcinoma. Cancers.

[16] Han X, Jiang H, Qi J (2020). Novel lncRNA UPLA1 mediates tumorigenesis and prognosis in lung adenocarcinoma. Cell Death Dis.

[17] Li J, Han Y, Zhang H (2019). The m6A demethylase FTO promotes the growth of lung cancer cells by regulating the m6A level of USP7 mRNA. Biochem Biophys Res Commun.

[18] Jin D, Guo J, Wu Y (2019). m^6^A mRNA methylation initiated by METTL3 directly promotes YAP translation and increases YAP activity by regulating the MALAT1-miR-1914-3p-YAP axis to induce NSCLC drug resistance and metastasis. Journal of hematology & oncology.

[19] Wang T, Kong S, Tao M (2020). The potential role of RNA N6-methyladenosine in Cancer progression. Mol Cancer.

[20] Qian X, Yang Z, Qiu Q (2021). LCAT3, a novel m6A-regulated long non-coding RNA, plays an oncogenic role in lung cancer via binding with FUBP1 to activate c-MYC. J Hematol Oncol.

[21] Liu J, Liu ZX, Wu QN (2020). Long noncoding RNA AGPG regulates PFKFB3-mediated tumor glycolytic reprogramming. Nature Communications.

[22] Vargas RE, Wang W (2020). Significance of long non-coding RNA AGPG for the metabolism of esophageal cancer. Cancer Commun (Lond).

[23] The Cancer Genome Atlas Research Network (2014). Comprehensive molecular profiling of lung adenocarcinoma. Nature.

[24] Campbell J, Alexandrov A, Kim J (2016). Distinct patterns of somatic genomealterations in lung adenocarcinomas and squamous cell carcinomas. Nat Genet.

[25] Rudi K, Sekelja M (2013). High or low correlation between co-occuring gene clusters and 16S rRNA gene phylogeny. FEMS Microbiol Lett.

[26] Gutschner T, Hämmerle M, Eissmann M (2013). The noncoding RNA MALAT1 is a critical regulator of the metastasis phenotype of lung cancer cells. Cancer Res.

[27] Kleczko EK, Kwak JW, Schenk EL (2019). Targeting the Complement Pathway as a Therapeutic Strategy in Lung Cancer. Front Immunol.

[28] Travis WD, Brambilla E, Noguchi M (2011). International association for the study of lung cancer/american thoracic society/european respiratory society international multidisciplinary classification of lung adenocarcinoma. J Thorac Oncol.

[29] Relli V, Trerotola M, Guerra E (2019). Abandoning the Notion of Non-Small Cell Lung Cancer. Trends Mol Med.

[30] Li J, Li Z, Zhang W (2017). LncRNA-ATB: An indispensable cancer-related long noncoding RNA. Cell Prolif.

[31] Wu X, Sui Z, Zhang H (2020). Integrated Analysis of lncRNA-Mediated ceRNA Network in Lung Adenocarcinoma. Front Oncol.

[32] Feng T, Zhang Q, Li Q (2020). LUAD transcriptomic profile analysis of d-limonene and potential lncRNA chemopreventive target. Food Funct.

[33] Dong HX, Wang R, Jin XY (2018). LncRNA DGCR5 promotes lung adenocarcinoma (LUAD) progression via inhibiting hsa-mir-22-3p. J Cell Physiol.

[34] Mao W, Li T (2020). LncRNA MACC1-AS1 Promotes Lung Adenocarcinoma Cell Proliferation by Downregulating PTEN. Cancer Biother Radiopharm.

[35] Tang W, Yu X, Zeng R (2020). LncRNA-ATB Promotes Cisplatin Resistance in Lung Adenocarcinoma Cells by Targeting the miR-200a/β-Catenin Pathway. Cancer Manag Res.

[36] Sun T, Wu R, Ming L (2019). The role of m6A RNA methylation in cancer. Biomed Pharmacother.

[37] Ma S, Chen C, Ji X (2019). The interplay between m6A RNA methylation and noncoding RNA in cancer. J Hematol Oncol.

[38] Sheng H, Li Z, Su S (2020). YTH domain family 2 promotes lung cancer cell growth by facilitating 6-phosphogluconate dehydrogenase mRNA translation. Carcinogenesis.

[39] Zhao Z, Cai Q, Zhang P (2021). N6-Methyladenosine RNA Methylation Regulator-Related Alternative Splicing (AS) Gene Signature Predicts Non-Small Cell Lung Cancer Prognosis. Front Mol Biosci.

[40] Lin S, Choe J, Du P (2016). The m(6)A Methyltransferase METTL3 Promotes Translation in Human Cancer Cells. Mol Cell.

[41] Wanna-Udom S, Terashima M, Lyu H (2020). The m6A methyltransferase METTL3 contributes to Transforming Growth Factor-beta-induced epithelial-mesenchymal transition of lung cancer cells through the regulation of JUNB. Biochem Biophys Res Commun.

[42] Yu H, Zhang Z (2021). ALKBH5-mediated m6A demethylation of lncRNA RMRP plays an oncogenic role in lung adenocarcinoma. Mamm Genome.

[43] Xue L, Li J, Liu D (2021). m(6) A transferase METTL3-induced lncRNA ABHD11-AS1 promotes the Warburg effect of non-small-cell lung cancer. J Cell Physiol.

